# Leaf form diversity and evolution: a never-ending story in plant biology

**DOI:** 10.1007/s10265-024-01541-4

**Published:** 2024-04-09

**Authors:** Hokuto Nakayama

**Affiliations:** Graduate School of Science, Department of Biological Sciences, The University of Tokyo, Science Build. #2, 7-3-1 Hongo Bunkyo-ku, Tokyo, 113-0033 Japan

**Keywords:** Evo-Devo, Evolution, Leaf, Leaf development, Leaf form diversity

## Abstract

Leaf form can vary at different levels, such as inter/intraspecies, and diverse leaf shapes reflect their remarkable ability to adapt to various environmental conditions. Over the past two decades, considerable progress has been made in unraveling the molecular mechanisms underlying leaf form diversity, particularly the regulatory mechanisms of leaf complexity. However, the mechanisms identified thus far are only part of the entire process, and numerous questions remain unanswered. This review aims to provide an overview of the current understanding of the molecular mechanisms driving leaf form diversity while highlighting the existing gaps in our knowledge. By focusing on the unanswered questions, this review aims to shed light on areas that require further research, ultimately fostering a more comprehensive understanding of leaf form diversity.

## Introduction

Plants have a wide range of leaf shapes. In many cases, morphological diversity is thought to reflect adaptations to various environmental factors, such as temperature, light, and water availability (Tsukaya 2018). In addition to the leaf form diversity observed in nature, diversity is also found in many cultivars of horticultural plants and crops (Nakayama et al. 2022). In extreme cases, morphological diversity is observed in a single plant; heteroblasty and heterophylly are phenomena of leaf morphological alterations within a single individual, during the different developmental stages, or in response to the surrounding environment, respectively (Nakayama et al. 2017; Poethig 2010; Zotz et al. 2011). Therefore, leaf form diversity is observed at different levels, such as a single individual, inter-species, among cultivars, and intraspecies. Understanding the molecular mechanisms underlying leaf morphological diversity is of great significance in the field of plant biology, particularly in terms of evolutionary, developmental, ecological, and physiological biology. Numerous studies have disclosed the genetic factors and molecular mechanisms influencing leaf form diversity since the 2000s (Bharathan et al. 2002; Blein et al. 2008; Garcês et al. 2007; Richardson et al. 2021; Vlad et al. 2014; Whitewoods et al. 2020) after the basic molecular mechanisms of leaf development were revealed using a few model plant species such as *Arabidopsis thaliana* (Maugarny-Calès and Laufs 2018). However, although significant progress has been made in the field of leaf morphological diversity, particularly regarding the basic molecular framework for the regulation of leaf complexity, many other questions remain unanswered.

This review aims to outline the existing knowledge of the molecular mechanisms underlying leaf morphological diversity at different levels and reveal any unsolved questions and challenges in the field. Fortunately, there have been numerous review articles published in recent years regarding leaf development and molecular mechanisms (Conklin et al. 2019; Maugarny-Calès and Laufs 2018; Tsukaya 2021), therefore, I will not delve into details about the molecular mechanisms of basic leaf development. Instead, this review focuses on summarizing what has not yet been clarified. This may help highlight areas where research is required to deepen our comprehensive understanding of leaf form diversity and I believe that this review will help guide further development in this field.

## Leaf morphology between species and cultivars

**Fig. 1:**
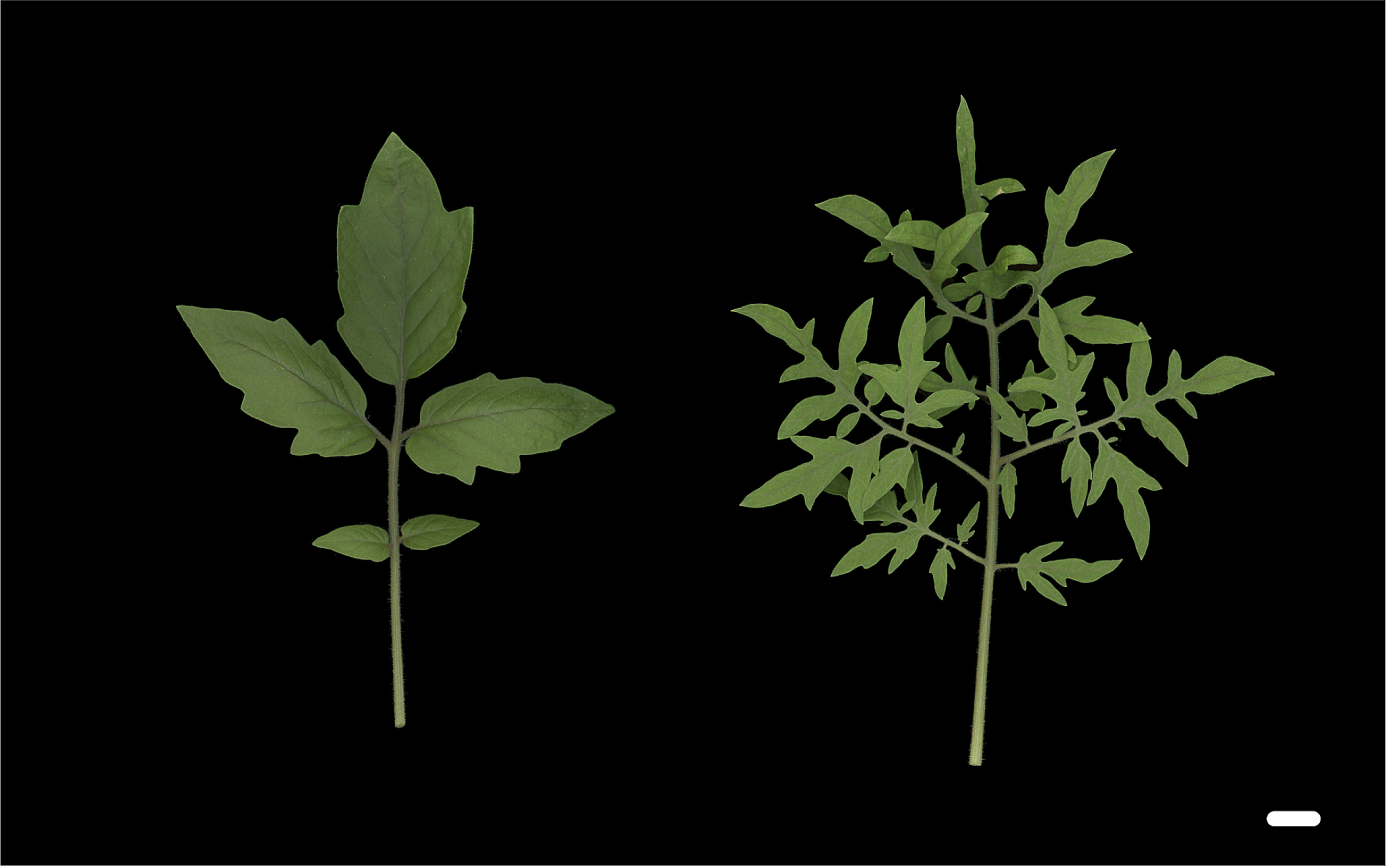
Leaf form variation of tomatoes. Left: *Solanum lycopersicum* cv. M82; right: an heirloom tomato “Silvery Fir Tree.” Bar = 1 cm

A key transcription factor in leaf morphological diversification is the class I KNOTTED-like homeobox (KNOX1). Maize *knotted1* (*kn1*), a KNOX1 gene, was the first homeobox gene found in plants (Hake et al. 1993), and detailed studies using several model plants such as *Arabidopsis thaliana* (Arabidopsis) and *Zea mays* (maize) have revealed that it is involved in the formation and maintenance of the shoot apical meristem (SAM) (Jackson et al. 1994; Long et al. 1996). Recent studies have revealed that SHOOT MERISTEMLESS (STM), a mobile KNOX1 protein, trafficking among cells, is indispensable not only for the maintenance and differentiation of shoot stem cells but also for axillary meristem formation, meristem size, and organ boundaries (Balkunde et al. 2017; Kitagawa et al. 2022; Liu et al. 2018). Moreover, the *kn1* gene also has important functions in leaf morphology, as shown by its original isolation as a maize leaf mutant (Hake et al. 1993). Subsequent studies on tomatoes, a well-studied model plant for leaf development, showed that overexpression of the KNOX1 gene in the leaf primordium increased leaf complexity (Chen et al. 1997). Indeed, there are several examples of KNOX1 contributing to differences in the leaf morphology in wild and heirloom tomatoes. A Galapagean tomato, *Solanum galapagense*, exhibits higher leaf complexity. It has a single nucleotide deletion in the promoter of *PETROSELINUM* (*PTS*), a short KNOX1 gene that lacks a DNA-binding domain, leading to *PTS* ectopic expression in young leaves. PTS inhibits the KNOX1 protein interaction with BIPINNATA (BIP) and the KNOX1–BIP complex regulates leaf complexity by modulating KNOX1 expression. Higher expression of KNOX1 in the leaf primordia due to a mutation in the *PTS* promoter leads to increased leaf complexity in this Galapagean tomato (Kimura et al. 2008). Another study demonstrated that Silvery Fir Tree (SiFT), a Russian heirloom tomato showing increased leaf complexity, had a single nucleotide deletion in the *BIP* gene, leading to a higher expression of KNOX1 in the leaves (Fig. 1). Comparative genomics indicated that the *bip* mutation in SiFT likely arose *de novo*, is unique to SiFT, and is not introgressed from other tomato genomes (Nakayama et al. 2021). In addition, several studies have shown that KNOX1 is involved in leaf complexity in different plant species (Wang et al. 2022).

Simple and compound leaves, the most basic classifications of plant leaves, can also be regarded as having differences in complexity. Simple and compound leaves are thought to share common gene regulatory networks and compound leaves undergo repeated leaflet organogenesis (Tsukaya 2014). In this context, KNOX1 remains a key player in facilitating the formation of different leaf shapes between simple and compound leaves. A previous comparative study of *Cardamine hirsuta* (Brassicaceae) and Arabidopsis demonstrated that *STM* is necessary for leaflet morphogenesis and that the cis-regulatory divergence in two KNOX1 genes, *STM* and *BREVIPEDICELLUS* (*BP*), is associated with differences in leaf shape between them (Hay and Tsiantis 2006). Additionally, a more recent study using loss- and gain-of-function mutants and transgenics suggested that *stm* mutants are more pleiotropic than *bp* mutants. Yet, the *BP* genomic locus from *C. hirsuta* is sufficient to modify the Arabidopsis leaf shape to a much greater extent than the *C. hirsuta STM* locus. Therefore, the authors concluded that although *BP* has a lesser pleiotropic effect on plant development, cis-regulatory differences in the *BP* locus between *C. hirsuta* and Arabidopsis are also sufficient to change leaf shape (Rast-Somssich et al. 2015). Regarding the Arabidopsis leaf shape, a previous study on several species in the genus *Arabidopsis* demonstrated that the evolution of its unlobed leaf form involved the loss of STM expression in the leaf primordia, and cis-regulatory divergence contributed to this process (Piazza et al. 2010). However, considering broader taxa, factors other than KNOX1 are also known to be involved in the evolution of leaf complexity. For instance, it appears that some simple-leafed species (e.g., *Lepidium oleraceum* and *Pimpinella anisum*) have a cryptic compound developmental program in the early stages of initiation, which is then secondarily modified to generate a simple morphology (Bharathan et al. 2002; Champagne and Sinha 2004; Champagne et al. 2007). This indicates that KNOX1 expression in the leaf primordia is not necessarily associated with compound leaves. In Fabaceae, compound-leafed species belonging to the inverted repeat–lacking clade use *UNIFOLIATA* (*UNI*), an ortholog of the floral regulator LEAFY/FLORICAULA (LFY/FLO), instead of KNOX1 to regulate compound leaf development (Hofer et al. 1997). In *Medicago truncatula* (Fabaceae), *PINNATE-LIKE PENTAFOLIATA1*, a *BIP* ortholog, negatively regulates the transcription of the *LFY* ortholog *SINGLE LEAFLET1* through direct protein–DNA interactions (He et al. 2020). Moreover, a series of studies on *Cardamine hirsuta* demonstrated that *REDUCED COMPLEXITY* (*RCO*), a class I HD-ZIP, suppresses local leaf growth by regulating multiple cytokinin (CK)-related genes (Hajheidari et al. 2019; Vlad et al. 2014). A previous study showed that the RCO and ChLMI1 in *C. hirsuta* are functionally equivalent in a developmental context (Vlad et al. 2014). These studies have revealed the molecular mechanisms of compound leaf development and its evolutionary trajectory.

Because KNOX1 is a well-known and crucial factor in plant development and regulation of leaf complexity, it tends to be selected first when studying leaf morphological diversity using candidate gene approaches, and its contribution may be overestimated. Further studies with diverse plant taxa will reveal the true generality and centrality of KNOX1 and its interactor systems, which are involved in the regulation of leaf complexity, including compound leaf formation.

## Leaf morphology within a single species/individual: heteroblasty and heterophylly

**Fig. 2:**
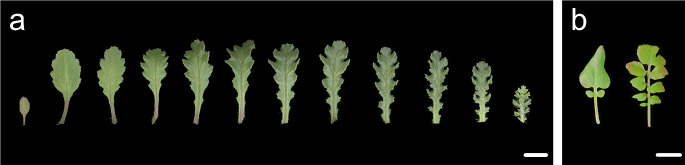
Heteroblasty and heterophylly. (**a**) An example of heteroblasty in *Senecio vulgaris*. The oldest (cotyledons) is on the left, and the youngest leaf is on the right. (**b**) An example of heterophylly in *Rorippa aquatica*. Left: 25 ˚C; Right: 20 ˚C. Bars = 1 cm

It is not only among different species that the leaf shape is diverse. In many plants, the leaf shape is extremely plastic even in a single individual. Regardless of the conditions, leaf shape changes along with the developmental stages. This phenomenon is called heteroblasty and refers to conspicuous morphological changes in leaves throughout the lifecycle of plants (Zotz et al. 2011; Fig. 2a). In Arabidopsis, the vegetative phase of leaf development can be divided into juvenile and adult phases. Differences between the two phases are characterized by the initiation of trichomes on the abaxial surface of the leaf, production of serrations on the leaf margin, leaf blade size, and leaf aspect ratio (Poethig 2010). Additionally, a recent study using confocal microscopy, forward and reverse genetics, and simulations suggested that the longitudinal expansion of giant cells in the leaf primordia, accompanied by a prolonged cell proliferation phase in their vicinity, is also associated with the establishment of adult leaf shape (Tang et al. 2023). The basic framework of the regulatory mechanisms of heteroblasty can be explained by the interaction between miR156 and miR156-targeted SQUAMOSA PROMOTER protein-binding protein-like (SPLs) transcription factors. SPLs are master regulators of temporal changes in leaf shape, and their mRNA is regulated by miR156, which is abundant in seedlings and gradually decreases with plant age (Cheng et al. 2021; Wang et al. 2008; Wu and Poethig 2006; Xu et al. 2016). Eleven SPL genes have been identified in the Arabidopsis genome (Xing et al. 2013) and previous studies have revealed redundant and specific roles of the SPLs in heteroblasty (Usami et al. 2009; Xu et al. 2016). Increased levels of *SPL2*, *9*, *10*, *11*, *13*, or *15* promote the development of adult leaf traits (Shikata et al. 2009; Wang et al. 2008). Among them, *SPL10* possibly regulates leaf heteroblasty by activating genes involved in the cell cycle and cell wall loosening, which leads to shape-shifting in leaves over time (Tang et al. 2023). Interestingly, this heteroblasty seems to be associated with a leaf form diversity, namely swollen thorn syndrome in ant-acacias (e.g., *Vachellia collinsii*), which is known for its specialized traits that provide ants with food and shelter (Leichty and Poethig 2019). As this plant grows, it begins to develop Beltian bodies, which are small structures containing nutrient-rich substances that can be food for ants and shelters on leaflets and leaf bases. Gene expression and developmental analyses suggested that this syndrome is associated with a temporal decline in microRNA miR156 and a corresponding increase in SPLs. Additionally, a comparison of closely related species indicated that this syndrome evolved by co-opting a preexisting age-dependent program (Leichty and Poethig 2019).

Studies on plants other than Arabidopsis have revealed some of the molecular mechanisms underlying heteroblasty. A comparative study of the closely related species Arabidopsis and *C*. *hirsuta* revealed that the *LFY* ortholog influenced heteroblasty in *C. hirsuta* (Monniaux et al. 2017). Nutrients also influence plant growth, maturation, and aging. For instance, sugar promotes vegetative phase changes in Arabidopsis by repressing the expression of miRNAs (Yang et al. 2013), and glucose may mediate adult leaf characteristics during *de novo* shoot organogenesis in *Passiflora edulis* by modulating miR156-targeted *PeSPL9* expression levels during the early stages of shoot development (Silva et al. 2019). These studies indicate that although the basic framework is well understood in Arabidopsis and its closely related species, the regulatory mechanism of heteroblasty is more complex as a whole and it would be useful to study not only Arabidopsis but also other plants, to capture and understand the whole system.

The surrounding environment is also an important factor that determines leaf morphology. This phenomenon is known as phenotypic plasticity (West-Eberhard 2003; Zotz et al. 2011; Fig. 2b). Phenotypic plasticity that leads to leaf form alterations in response to environmental conditions is called heterophylly (reviewed by Nakayama et al. 2017). Recent progress in this field has been remarkable, and, unlike heteroblasty, studies on non-model plants have been responsible for this significant progress (Kim et al. 2018; Koga et al. 2021; Kuwabara et al. 2003; Kuwabara and Nagata 2006; Li et al. 2020; Nakayama et al. 2014). These studies have revealed that, in heterophylly, rather than a mechanism that is common to all species, a mechanism that is specific to each species is more important. For instance, *Rorippa aquatica* (Brassicaceae) is a perennial herbaceous and semiaquatic plant in North America that shows distinct heterophylly under submerged (highly complex leaves) and terrestrial (less complex to simple leaves) conditions (Nakayama et al. 2014). A previous study on *R. aquatica* demonstrated that the expression level of a KNOX1 ortholog changes in response to changes in the surrounding environment, leading to changes in plant hormone concentrations, such as gibberellins (GAs) and CKs, in leaf primordia (Nakayama et al. 2014). GAs and CKs are involved in cellular differentiation and proliferation, respectively. In the case of *Hygrophila difformis* (Acanthaceae), previous studies have demonstrated that KNOX1 and CKs are key to the regulation of heterophylly (Li et al. 2017, 2020). These two species exhibited remarkable changes in leaf complexity between the submerged and terrestrial conditions as heterophyllous phenotypes. In these species, hormonal regulation through changes in the KNOX1 expression may be one of the main mechanisms of heterophylism in terms of alterations in leaf complexity. However, a study with *Ranunculus trichophyllus* (Ranunculaceae), an amphibious plant, suggested that abscisic acid (ABA) and ethylene regulate terrestrial and aquatic leaf development, respectively (Kim et al. 2018). Additionally, more recent studies on *Callitriche palustris* (Plantaginaceae) have emphasized that changes in leaf epidermal cell shape are also important for the regulation of heterophylly (Koga et al. 2021). In this species, epidermal cells display a jigsaw puzzle shape in the aerial leaves (ovate leaf shape). In contrast, the submerged leaves (elongated leaves) have a simpler and more elongated shape. The association between leaf morphology and epidermal cell shape in heterophylly has long been noted (Kuwabara and Nagata 2006; Kuwabara et al. 2003), and a study using *C*. *palustris* may provide insights into this association at the genetic regulatory level. Taken together, the genetic mechanisms underlying heterophyllous leaf-form alterations are diverse. This may be because heterophylly has evolved independently multiple times. On the other hand, it is interesting to note that phytohormones are involved in the regulation of heterophylly in many aquatic and amphibious plants. Ethylene and abscisic acid (ABA) regulate heterophylly in several plants (Kim et al. 2018; Koga et al. 2021; Kuwabara et al. 2003; Li et al. 2017; Sato et al. 2008). Ethylene is known to spontaneously accumulate in the plant body because of the limitation of gas exchange under submerged conditions (Davis and McKetta 1960), and ABA is involved in the water stress response (Kuromori et al. 2018). Moreover, an ethylene response has been observed in a wide range of angiosperms (Van de Poel and de Vries 2023), and the ABA pathway is conserved in green plant lineages (Takezawa et al. 2011). Therefore, it seems reasonable that these hormones are utilized to trigger submergence responses such as heterophylly. However, the details of the mechanisms by which phytohormone concentrations and susceptibilities change in response to environmental cues and how they translate into the regulation of heterophylly control have not been clarified. Therefore, the study of heterophylly will provide an opportunity not only to reveal the mechanisms of leaf morphological diversity but also to understand the diversity of molecular mechanisms involved in hormone regulation that cannot be revealed by model plants alone.

## Specialized leaves

**Fig. 3:**
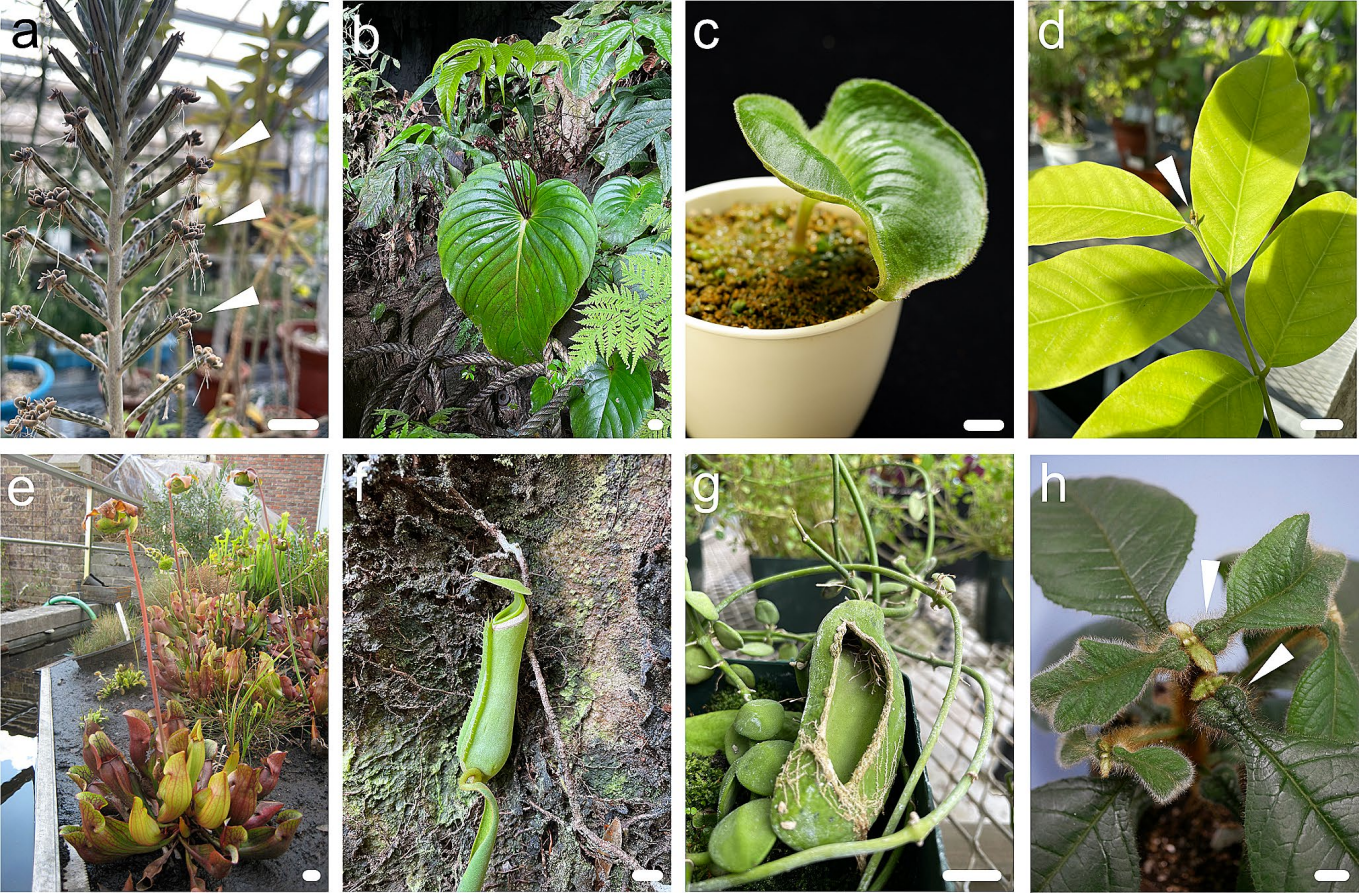
Specialized leaves. (**a**) *Kalanchoe delagoensis*: a *Kalanchoe* species that makes tiny plantlets on leaves. The white arrowheads indicate plantlets. (**b**) *Monophyllaea glauca* with an indeterminate macrocotyledon. (**c**) *Monophyllaea glabra*. Photograph courtesy of Dr. Shunji Nakamura. (**d**) A compound leaf of *Guarea guidonia*. The white arrowhead indicates the leaf apical meristem, which produces leaflets. (**e**) *Sarracenia purpurea*: a pitcher plant in the genus *Sarracenia*. (**f**) *Nepenthes albomarginata*. (**g**) Leaves of *Dischidia rafflesiana*, offering accommodation to ants. The largest leaf in this figure has a window made so that the inside can be seen. The roots are seen penetrating the leaf. (**h**) *Callicarpa saccata* which is an ant plant. Photograph courtesy of Prof. Hirokazu Tsukaya. The white arrowhead indicates domatia. Bars = 1 cm

Some plant leaves undergo complex deformations to achieve special functions; such plants have also been the subject of extensive research. For instance, some species in the genus *Kalanchoe* (Crassulaceae) produce tiny plantlets that drop from the edges of their leaves (Fig. 3a). However, the molecular mechanisms underlying plantlet formation at the leaf edges remain unclear. Using *Kalanchoe diagremontiana*, detailed developmental observations, expression analysis of orthologs involved in shoot organogenesis (*STM*) and embryogenesis (*LEAFY COTYLEDON 1*: *LEC1*), and transgenic experiments demonstrated that the expression of *STM* orthologs in leaves was essential for producing plantlets on the leaf edge in *K*. *diagremontiana* (Garcês et al. 2007). Although the LEC1 protein is truncated in this species and its function is unclear, it is expressed on the leaf edge, suggesting that an embryo-like program is also involved in plantlet development. Although further experiments are necessary to determine whether the origin of the truncated *LEC1* protein is causal or consequential in plantlet formation, a combination of changes in these genes is thought to allow the *Kalanchoe* leaf sinus to produce embryo-like plantlets (Garcês et al. 2007). Plants in the genus *Monophyllaea* (Gesneriaceae) do not produce new organs in the shoot during the vegetative phase, whereas one of the cotyledons grows indeterminately, making the plants anisocotyledonous (Fig. 3b, c). Although some plants with indeterminately growing leaves are known in the extant taxa such as *Monophyllaea*, *Guarea* (Fig. 3d), and Welwitschia, the details of their unique developmental processes and organ identities are not clear in any plants. A previous study on *M*. *glabra* suggested that this anisocotyledon phenotype may be the result of biased auxin concentrations between the two cotyledons and the subsequent CK levels, a hormone known to induce cell proliferation (Kinoshita and Tsukaya 2022). This indeterminate growth of macrocotyledons, larger cotyledons, and the mechanism that prevents the emergence of new organs in *Monophyllaea* provides an opportunity to gain a deeper understanding of the general growth processes in plants, particularly the regulation of determinate growth. Detailed anatomical and developmental analyses suggested that in *M*. *glabra*, macrocotyledons have different types of meristems: the groove meristem (GM), which is located at the junction between the macrocotyledon and petiolode (Jong 1970; Jong and Burtt 1975), is thought to correspond to the SAM, and the basal meristem (BM), which is located in the basal part of the lamina, contributes to lamina growth by active cell division. The expression patterns of orthologs of *STM* and *ANGUSTIFOLIA3*/*GRF-INTERACTING FACTOR1*, a transcription coactivator that regulates cell proliferation and adaxial/abaxial (ad/ab) patterning (Horiguchi et al. 2005, 2011), in macrocotyledons indicate that the indeterminate growth of macrocotyledons results from a combination of both shoot and leaf characteristics in typical plants. This possibly contributes to the unusual morphology and developmental patterns of *M*. *glabra* (Kinoshita et al. 2020).

So far, I have discussed changes in “flat” leaves; however, some leaves are more complex, in particular, those that exhibit 3D shapes. In these more complex leaf shapes, it is an interesting question whether there are special mechanisms to create these complex 3D shapes, or whether they can be understood by a combination of relatively simple mechanisms. A previous study on *Sarracenia purpurea* (Sarraceniaceae; Fig. 3e), a carnivorous plant with pitcher leaves (Fig. 3f), showed that the establishment of cell division direction at the tissue-specific level, rather than the modification of ad/ab polarity in leaves, is crucial for the development of pitcher-shaped leaves (Fukushima et al. 2015). A recent study using *Utricularia gibba* (Lentibulariaceae), a carnivorous aquatic plant, showed that regional identities that modify growth rates oriented by two orthogonal polarity fields are necessary to provide a more complex leaf shape (Lee et al. 2019; Whitewoods et al. 2020). In this study, a model was developed that could generate diverse leaf forms and account for the evolutionary origins of cup-shaped leaves through simple alterations in gene expression (Whitewoods et al. 2020). Similar processes may be happening in the bullate leaves of *Dischidia* plants (Fig. 3g). Similarly, a study focusing on the domatium, a cavity within the structure of plants inhabited by ants and mites, suggested that excessive cell proliferation at the basal part of the leaf blade warps the shape of the blade and disturbs the development of the proximodistal axis, leading to the generation of domatia in *Callicarpa saccata* (Lamiaceae; Sarath et al. 2020), which is a mymecophytic species (Nakashima et al. 2016; Fig. 3h). These studies indicate that relatively parsimonious cues can generate diverse leaf forms via changes in the orientation of cell division and/or gene expression involved in the establishment of polarity fields. Additionally, recent studies have revealed the importance of cell proliferation position: a study on the relationship between cell division patterns and the final organ shape of sepals and petals, which are the same lateral organs as leaves, revealed that the difference in the location of cell division associated with the spatial *AN3* expression pattern mainly contributes to the final organ shape (Kinoshita et al. 2022). This difference may indicate that cell division patterns and the area in which cell division occurs contribute differently to the 2D and 3D morphologies of leaves. However, the detailed developmental patterns, impact of cell division patterns, and position of cell division area in leaf form diversification still need to be clarified in further studies to evaluate each contribution.

In Arabidopsis, leaf meristematic tissues can be divided into two types, long-lived plate meristems (PM) and short-lived marginal meristems (MM), which are controlled by two different pathways (Tsukaya 2021). Although leaf morphology is thought to depend on two meristematic tissues, even in Arabidopsis, the contributions of PM and MM to leaf morphogenesis are unknown. For example, although it has been reported that the transient cell proliferation activity of MM is due to the biased expression of the CIN-TCP-NGA pathway components along the proximodistal axis and that at least part of this effect is mediated by the direct activation of transcription of the KIP-related proteins which are cell cycle inhibitor genes (Rath et al. 2022), little is known about the factors that determine the length of the meristematic activity period. Additionally, the factors that regulate the position of the meristematic regions within a leaf are unknown. Moreover, studies on non-model plants have indicated that other meristematic tissues contribute to the diversification of leaf morphology in some plants. For instance, studies on *Juncus* (Juncaceae) unifacial leaves that lack the adaxial fate of leaf lamina have shown that thickening meristem also plays an important role in leaf morphology, and an ortholog of *DROOPING LEAF* (*DL*), a CRABS CRAW–type YABBY gene, contributes to the thickening growth along the axis perpendicular to the direction of the expansion of bifacial leaves (Yamaguchi et al. 2010). This was sustained by active cell divisions distributed diffusely throughout the cross-sectional area of the leaf blade (Yin and Tsukaya 2019). A subsequent study revealed that the proper placement of auxin induces *DL* ortholog expression, leading to initial leaf flattening (Nukazuka et al. 2021). A similar mechanism was reported in other independently acquired unifacial leaves, and the importance of thickening meristem is worth emphasizing (Golenberg et al. 2023). Therefore, understanding each leaf meristem in detail and recapturing the complexity and specialization of leaf morphology through changes in these will help us understand the process of morphological change and the molecular mechanisms behind them more precisely. In Arabidopsis petals, cell proliferation mainly occurs in the distal region (Kinoshita et al. 2022), and is considered a good tool for studying the relationship between organ morphology and mitotic regions. The mechanisms underlying the morphological diversification of not only leaves but also petals, which have high morphological diversity and are the same lateral organ as leaves, may be useful in revealing the fundamental mechanism of morphological diversification in lateral organs, including leaves.

## Leaf-like organs

**Fig. 4:**
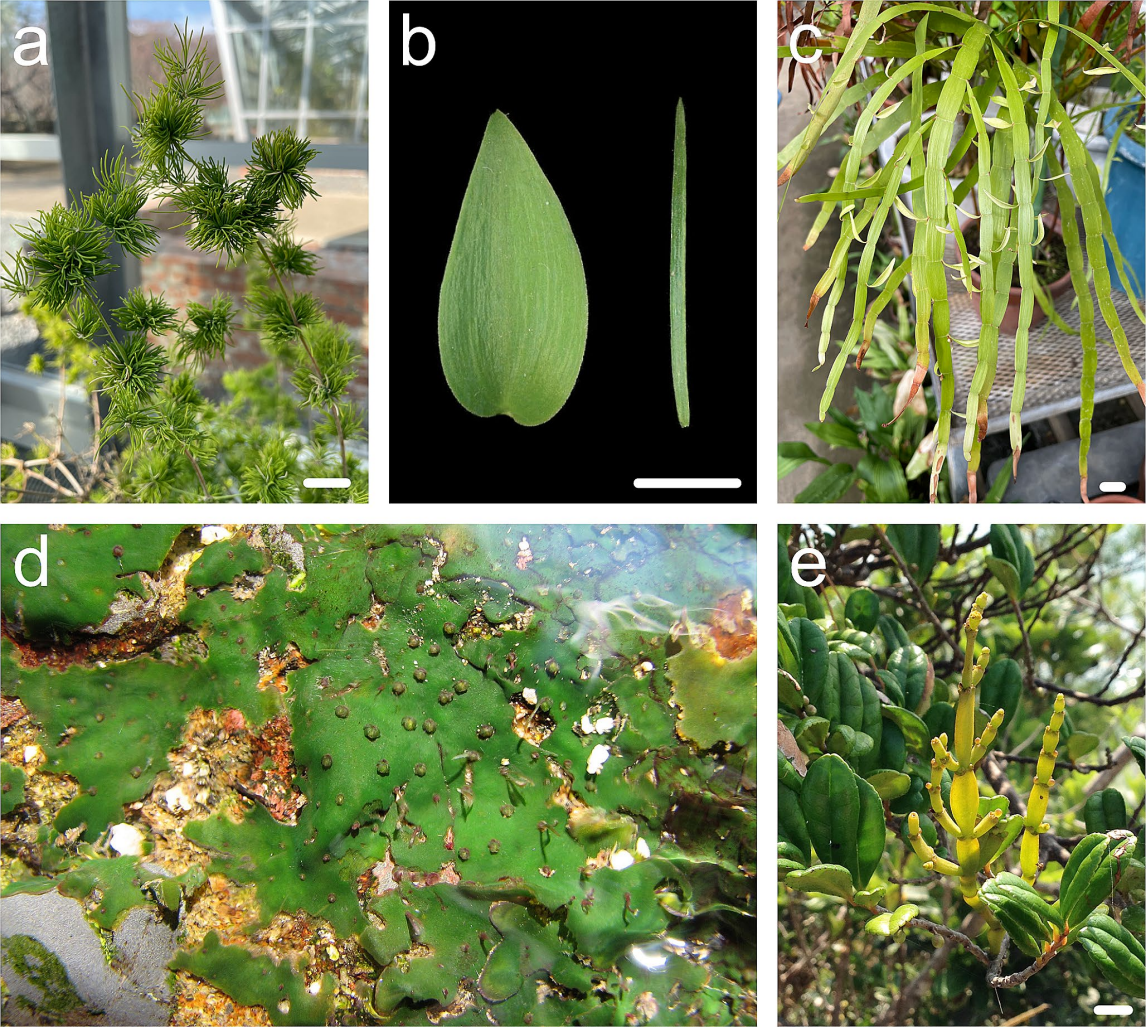
Flattened stems and roots. (**a**) *Asparagus myriocladus*. Note that the needle-like structures that look like leaves are all cladodes. (**b**) *Asparagus* cladodes. Left: *A. asparagoides*. Right: *A. cochinchinensis*. (**c**) *Muehlenbeckia platyclados*. Note that the flattened structures that look like leaves are phylloclades. (**d**) Branching lobed thalli of *Hydrobryum japonicum*. Photograph courtesy of Dr. Natsu Katayama. (**e**). *Korthalsella japonica* shoots grown on *Eurya emarginata*. Bars = 1 cm

Foliage leaves in the genus *Asparagus* are scale leaves that are reduced in size and do not appear to be fully functional as leaves. Instead, *Asparagus* plants have leaf-like organs called cladodes. Cladodes develop in the axil where a lateral shoot generally arises; however, they are flattened and leaf-like in shape (Fig. 4a b). Due to the unusual morphology and position of emergence, cladodes have received much attention from several aspects and their origin has been debated for a long time; Arber (1924) concluded that cladodes are prophylls of abortive lateral shoots in the axils based on their leaf-like morphology and anatomical features. However, since it is known that organ arrangement in plants is quite rigid, previous studies have concluded that cladodes are modified lateral branches based on their axillary position (Cooney-Sovetts and Sattler 1987; Kubitzki and Rudall 1998). A study using *A. asparagoides* with leaf-like cladodes showed that the anatomical features of the cladodes were similar to those of the leaf, except that the positions of the xylem and phloem were inverted in cladodes compared to foliage leaves (Nakayama et al. 2012a). Although KNOX1 expression was limited to the peripheral region of the cladode primordia and finally decreased, an ortholog of *ASYMMETRIC LEAVES1* (*AS1*) was also expressed in the central part of the primordia, suggesting that cladodes differ from true foliage leaves. Additionally, the orthologs involved in the establishment of the ad/ab polarities were expressed in a leaf-like manner. These data indicate that cladodes are modified axillary shoots that co-opt the leaf gene regulatory network, and this co-option allows the axillary shoots to establish ad/ab polarity and to be flattened and indeterminate (Nakayama et al. 2012a, b).

In addition to *Asparagus*, many other plants exhibit flattened stems. For instance, *Muehlenbeckia platyclados* (Polygonaceae) has phylloclades, a type of flattened stem that consists of multiple internodes (Fig. 4c). The cacti of the genus *Opuntia* (Cactaceae) also have phylloclades. Therefore, it is worth investigating whether these flattened stems are the result of convergent or parallel evolution. A previous study using *Ruscus aculeatus* (Asparagaceae) suggested that both *RaSTM* which is expressed in vegetative and reproductive shoot apices, and *RaYABBY2* which is highly expressed in leaves, were co-expressed in the phylloclade primordia (Hirayama et al. 2007), indicating that the *Ruscus* leaf developmental pathways may have been co-opted into the phylloclade developmental pathway, such as in *A*. *asparagoides* and that the acquisition of leaf-like structures in these species seems to be a result of parallel evolution.

Additionally, there are many other examples of leaf-like organs that are not originally leaves, such as *Hydrobryum japonicum*, in which the root becomes leaf-like (Fig. 4d), and *Korthalsella japonica* (Fig. 4e), in which the stem becomes leaf-like. Moreover, there are many other organs that are not originally leaves but have planarity and function like leaves. In *H*. *japonicum*, the expression of genes involved in the maintenance of SAM and stem cells, such as *STM* and *WUSCHEL*, are reduced at the shoot apex, and the *AS1* ortholog is expressed instead, resulting in the shoot apex being converted to a leaf and the shoot becoming determinate. The acquisition of determinate shoot formation is thought to have enabled Podostemaceae to expand and diversify further into torrential environments (Katayama et al. 2010). However, the details of root flattening remain unclear in *H*. *japonicum*. Although little research has been done on how leaf-like organs develop and evolve, the understanding of the “original” leaf developmental mechanism is progressing, and its application to these leaf-like organs is expected to make rapid progress and unveil the molecular mechanisms behind their evolutionary process in the future.

## Succulent leaf

**Fig. 5:**
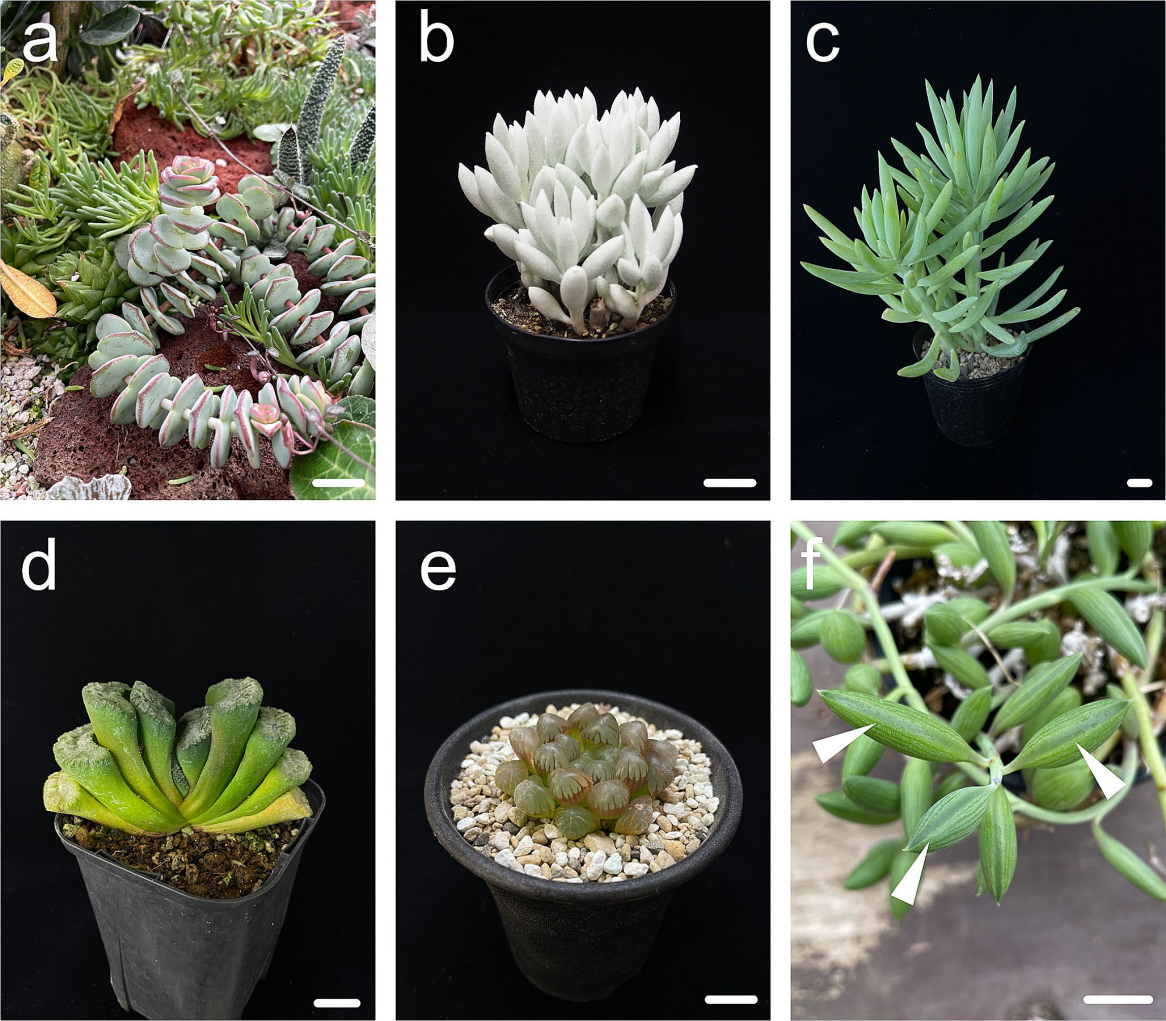
Various succulent plants. (**a**) *Crassula perforate*. (**b**) *Caputia tomentosa*, known as wooly senecio. (**c**) *Senecio antandroi*, a species of the genus *Senecio* endemic to Madagascar. (**d**) *Haworthia truncata*. The truncated tip has a leaf window. (**e**) *Haworthia cooperi*. A leaf window can be seen at the tip of the leaf. (**f**) *Curio herreanus*. The white arrowheads indicate leaf windows on the adaxial side. Bars = 1 cm

The examples of leaf diversification that I have provided so far are just part of the diversity that plants exhibit. Although the detailed developmental processes and molecular mechanisms are not clear, many plants exhibit interesting leaf morphologies. For instance, the succulent leaf, a type of leaf characterized by the presence of water-storage tissues that allow it to be temporarily independent of external water supply (Males 2017), presents an unusual leaf shape. In general leaves, morphological diversity is generated by deformation or alteration of the whole or a part of a flat leaf blade along the proximo-distal and mediolateral axes, and succulent leaves differ from other leaves in that they generate diversity through changes in the direction of leaf thickness (Fig. 5). Previous studies have suggested that cell enlargement begins with the loosening of the cell wall (Cosgrove 2016, 2023). This initial step induces the subsequent relaxation of wall stress, water uptake, and cell enlargement. Plant cell walls consist of cellulose microfibrils embedded in hydrophilic pectins and cellulose-binding hemicellulose (Cosgrove 2016, 2023). Recent studies have revealed that the wall strength, plasticity, and elasticity depend mostly on the stretching, straightening, and sliding of cellulose microfibril networks. In addition to the cell wall, vacuoles represent another component. In root cells, vacuolar occupancy increases from approximately 40% in meristematic cells to more than 85% in cells of the late elongation zone (Dünser et al. 2019). A recent study revealed that vacuolar enlargement is important for rapid cell expansion rates (Dünser et al. 2022), supporting the importance of vacuolation in plant cell enlargement. Recent data have also revealed that the receptor-like kinase, together with extracellular leucine-rich repeat extensins, senses cell wall properties (such as loosening) and subsequently impacts the intracellular expansion of the vacuole (Dünser et al. 2019). Because the cells of succulent leaves are known to be vacuolated, it would be interesting to determine whether these pathways are relevant in the enlarged cells of succulent leaves and, if so, how they contribute to shaping succulent leaves. In terms of increasing cell number, a study using Arabidopsis revealed that leaf thickness was enhanced under high light conditions compared to low light conditions through increases in both cell number and size in the direction of leaf thickness; this process was divided into two phases controlled by blue light and the amount of sugar supplied (Hoshino et al. 2019). Although this change did not cause dramatic leaf succulence in Arabidopsis, further elucidation of this mechanism in terms of an increase in cell number in the direction of thickness is important.

Another clue to consider regarding the association with succulent leaf development might be endoreduplication, which is a specialized cell cycle in which mitosis is bypassed, thereby producing cells with doubled genome ploidy. Endoreduplication is involved in cell enlargement, particularly in the epidermal pavement cells of Arabidopsis (Galbraith et al. 1991; Gendreau et al. 1997; Hülskamp et al. 1999; Katagiri et al. 2016; Kawade and Tsukaya 2017; Melaragno et al. 1993). Additionally, recent studies have revealed that endoreduplication is linked to cell expansion in the tomato pericarp, and the level of GA is a limiting factor in cell size and ploidy (Renaudin et al. 2023). Interestingly, previous studies have suggested that some succulent plants exhibit endoreduplication (De Rocher et al. 1990; Powell et al. 2020), and certain cells are known to expand in succulent leaves. Therefore, endoreduplication may be involved in the expansion of succulent leaves. However, even in Arabidopsis, the relationship between cell enlargement and endoreduplication and its molecular mechanisms are not fully understood, although a recent study emphasized that endoreduplication is a phenotype (result) of differentiated pavement cells and not a trigger of pavement differentiation (Dubois et al. 2023). Additionally, a recent study on tomato pericarps suggested that not only endoreduplication but also the position of the cell within the tissue is important for cell expansion (Tourdot et al. 2024). Therefore, further studies are required to determine the relationship between cell expansion and endoreduplication.

Finally, some succulents have leaf windows, also known as epidermal windows, and fenestrations in their leaves to allow light exposure (Fig. 5d–f). Leaf windows are seen at the tip or top part of the adaxial surface of leaves, allowing light to be captured even when the plant body is entirely below the soil surface in harsh environments, such as deserts, minimizing the exposure of the leaf surface area to desiccation due to intense heat (Egbert et al. 2008). There are different types of leaf windows, and it is interesting to understand how these windows have developed and evolved.

## Origin of leaves

**Fig. 6:**
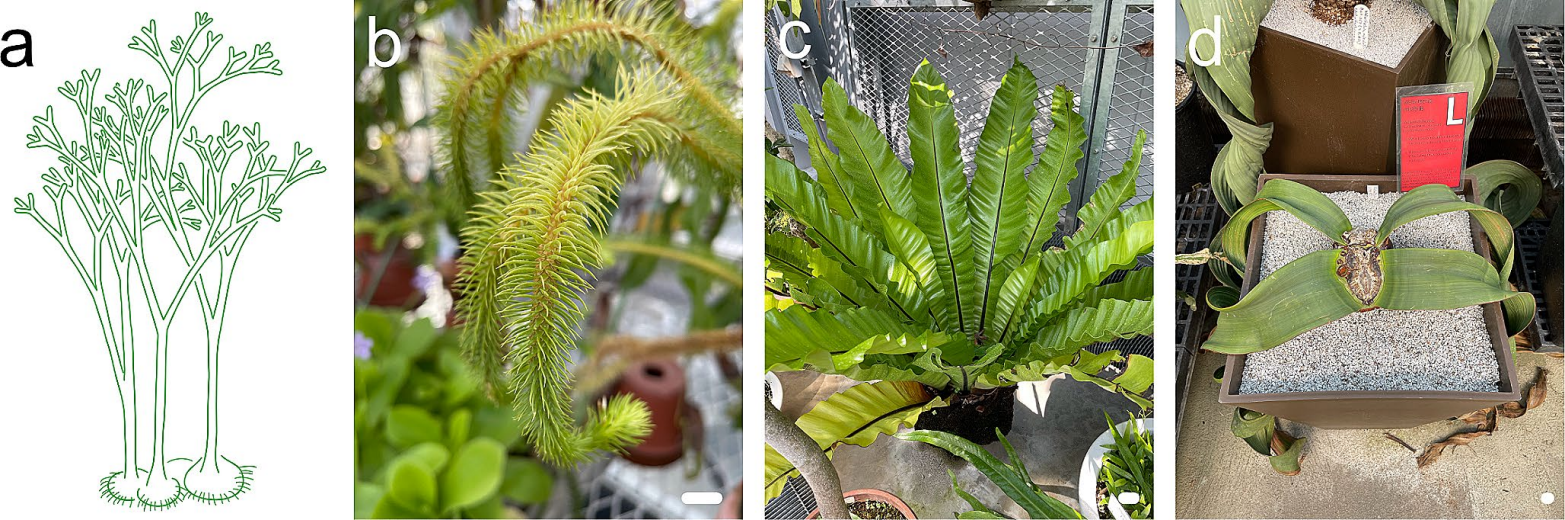
Leaves in different lineages in land plants. (**a**) Schematic reconstruction of *Horneophyton*, an extinct early plant. This figure is created based on Gifford and Foster (1989). (**b**) Leaves of *Phlegmariurus squarrosa*. (**c**) *Asplenium nidus*. (**d**) *Welwitschia mirabilis*. Bars = 1 cm in (**b**) and 2 cm in (**c**) and (**d**)

Thus far, we have discussed leaf morphological diversity and its evolution; however, the detailed process of leaf evolution and the underlying molecular mechanisms remain unknown. The fossil record suggests that the ancestors of land plants had a bifurcated branching (equal branching) system with no leaves and is thought to be similar to that of *Horneophyton* spp. (Fig. 6a). Thus, flattened leaves were acquired during the evolution of land plants (Romanova et al. 2023). The most famous hypothesis for the evolution of leaves from branching forms is the ‘telome theory’. This theory proposed that the transformative evolutionary processes of unequal branching “overtopping,” rearrangement of lateral branches into a single plane “planation”, and infilling of gaps between branches with laminar tissue “webbing” generated leaves (Zimmermann 1952). Fossil records that suggest distinct “webbing” have not been found to date, although “overtopping” and “planation” have been found in Trimerophyton. Additionally, there is no known process for the secondary fusion of multiple developed branches. Therefore, there is still no conclusive evidence supporting the telome theory. However, each process highlighted in the theory is thought to suggest a fundamental process in leaf development and evolution, as attempts are still being made to understand the evolutionary process of leaves by modifying the telome theory to take into account new findings (Harrison and Morris 2018; Vasco et al. 2016). One of the reasons why the process of leaf evolution is difficult to understand is that it has occurred multiple times during land plant evolution (Harrison and Morris 2018; Vasco et al. 2016). In addition to moss leaves, vascular plant leaves (lycophyte and horsetail leaves, and fern and seed plant fronds and leaves; Fig. 6b–d) have evolved multiple times independent of the branching shoot systems. Therefore, previously used terms such as “microphyll” and “megaphyll” are no longer used to emphasize that these leaves have been acquired in each lineage. In addition, our understanding of evolutionary origins of leaves is improving owing to the increased accuracy of phylogenetic relationships among existing and pre-existing land plants, and several studies have suggested that a specific set of genes (toolkit genes) tends to be used even in the development of leaves that have different origins (Harrison and Morris 2018). Moreover, recent genome analyses have suggested that many of the genes involved in plant development exist earlier on in the evolutionary process (Bowman 2022). This suggests that common toolkit genes (or part of the genes) may have been used in independently acquired leaves and raises new questions about what those genes were and how they changed and were used in each lineage. This question is perhaps one of the greatest mysteries related to leaves. The detailed evolutionary process of leaves has not yet been clarified, and further analyses on this subject are required.

## Conclusion

Many of the examples presented in this review are found not only in one specific lineage but also in multiple independent lineages. For example, indeterminate leaf growth, as observed in *M*. *glabra*, has also been observed in some *Guarea* species (Steingraeber and Fisher 1986; Fig. 3d) and *Welwitschia mirabilis*, a gymnosperm species (Fig. 6d). This indicates that understanding the molecular mechanisms underlying each leaf’s morphological diversification and evolutionary processes will shed light on whether they are the result of convergent or parallel evolution. The effect of KNOX1 on leaf morphology is one of the most interesting cases reported for multiple phenomena at various levels. Although a re-evaluation of its importance and centrality in leaf form diversification is needed, as mentioned above, leaf morphological diversity involving KNOX1 can be considered a “super-hotspot” and not just a hotspot. It is important to understand whether there are other such genes, and if so, what characteristics they have in the leaf developmental regulatory network. Additionally, some highly specialized leaves may be useful for studying complex adaptive traits. This is because it is still unclear how the complex adaptive traits evolve beyond the valley of adaptation, where they are adaptive in their completed state but not in the intermediate stages before completion and become disadvantageous for survival. Therefore, diversity in leaf morphology is not only fascinating in the realm of plant biology but also serves as a valuable research topic to shed light on issues that are relevant to all living organisms in terms of evolutionary aspects that can be adapted to the whole organism.

This review mainly focuses on the leaf lamina. However, the petiole, a stalk-like structure that attaches the leaf lamina to the stem, has not been extensively studied. Many leaves have this structure, and like other organs, petioles exhibit morphological diversity (Bell and Bryan 2008). For instance, in some species (e.g., *Acacia* spp.), the petiole flattens laterally into a photosynthetic organ, the phyllode (Dong and He 2017). Others have swollen petioles with cavities inhabited by ants, such as in *Piper cenocladum* (Tepe et al. 2009). Additionally, food bodies, which are structures that may contain proteins, lipids, or carbohydrates in different proportions and serve to maintain mutualistic relationships between the plant and ants, occur at specialized sites called trichilia at the swelling petiole-stem juncture in *Ceropia obtusa* (Bell and Bryan 2008; Marting et al. 2018). However, the basic molecular mechanisms underlying petiole development have not yet been studied, even in model plants. Therefore, understanding the molecular mechanisms of petiole development is another unpaved research field in leaf shape, and applying what we are about to uncover to understand the mechanisms underlying petiole morphological diversity will become more important in the future.

The latest estimate of the number of existing plants is more than 300,000 and this number is increasing every year (Christenhusz and Byng 2016); this suggests that studying the variety of leaf shapes and structures is an ongoing topic of plant research and this research field is just at its beginning. However, owing to recent advancements in RNA and DNA sequencing technologies and analysis pipelines, we can now study any plant at the molecular level, instead of being limited to model plants or closely related species. This will enable further research to be conducted on plants with interesting leaf morphologies. Therefore, for those interested in the morphology of organisms and their evolutionary processes, it can be argued that this is a never-ending subject of intellectual inquisitiveness and fun.
